# Importance of Serum Ferritin Level for Early Diagnosis and Differentiation in Patients with Kawasaki Disease with Macrophage Activation Syndrome

**DOI:** 10.3390/children8040269

**Published:** 2021-03-31

**Authors:** Da Eun Roh, Jung Eun Kwon, Hee Joung Choi, Yeo Hyang Kim

**Affiliations:** 1 Department of Pediatrics, School of Medicine, Kyungpook National University, Daegu 41405, Korea; ponyks1004@naver.com (D.E.R.); lovecello623@gmail.com (J.E.K.); 2 Division of Pediatric Cardiology, Kyungpook National University Children’s Hospital, Daegu 41404, Korea; 3 Department of Pediatrics, Keimyung University School of Medicine, Keimyung University Dongsan Hospital, Daegu 42601, Korea; joung-756@hanmail.net

**Keywords:** ferritin, fever, kawasaki disease, macrophage activation syndrome

## Abstract

We aimed to evaluate the utility of the serum ferritin level as an early screening test of Kawasaki disease with macrophage activation syndrome (KD-MAS). We analyzed the serum ferritin levels on the first day of admission and the clinical progress of patients diagnosed with complete or incomplete KD. Of the 158 patients, 5 were diagnosed with KD-MAS. Conjunctival injection was significantly more frequent in KD group (*p* = 0.035), although there were no significant differences in other clinical features. On the first day of admission, the serum ferritin level in the KD-MAS group was >500 ng/mL, which was higher than that in the KD group (*p* = 0.001). In the KD-MAS group, total bilirubin, triglyceride, and lactate dehydrogenase (LDH) were significantly higher, and erythrocyte sedimentation rate (ESR), total protein, albumin, and fibrinogen were significantly lower than the KD group (*p* < 0.05). Four patients were diagnosed with MAS within 7 days after admission, and 4 (80%) patients with KD-MAS survived. In conclusion, carrying out an early ferritin screening test is important in patients with principal clinical features that may suspect KD. We propose to include ferritin level in the primary laboratory test to differentiate between KD with and without MAS early.

## 1. Introduction

Macrophage activation syndrome (MAS) is an inflammatory phenomenon caused by excessive activation of T cells and macrophages, which is secondary to infections, malignancies, medications, or rheumatic diseases [1,2]. It is characterized by clinical symptoms such as persistent fever, hepatosplenomegaly, cytopenia, and organ dysfunction [3,4]. The most common disease combined with MAS was sJIA [1,5]. Diagnosis in the early stage is difficult for the following reasons: (1) clinical characteristics tend to overlap with those of other autoimmune disorders or sepsis and (2) clinical features and the change of biomarker levels do not occur simultaneously in the early stages of the disease. Therefore, the final diagnosis with MAS requires time.

Recently, it is also increasing in patients with MAS combined with KD (KD-MAS), and the incidence has been reportedly 1.1–1.9% [5,6,7]. In addition, it may occur in any stage of KD (acute, subacute or recovery stage), mostly simultaneously with KD [8,9]. KD-MAS tend to show high prevalence of nonresponsiveness to immunoglobulin (IVIG) treatment and a high risk of coronary artery complication, which can result in high morbidity and mortality rates [4,10,11]. However, MAS is clinically less recognizable in KD. To reduce morbidity and mortality rates of MAS, early recognition and diagnosis are essential.

Although there are no definite biomarkers for disease differentiations, the serum ferritin level has been used to screen patients at risk of developing MAS. Hyperferritinemia, especially with ferritin levels at >5000–10,000 ng/mL, is an important diagnostic biomarker of MAS [1,5].

We hypothesized that checking the ferritin level on initial laboratory test for KD will help identify KD-MAS risk patients in the acute phase of the disease. In the present study, we analyzed patients’ serum ferritin levels with conventional serologic test for KD on the first day of admission. Then, we assessed relations between the results of these laboratory test and clinical progress. We aimed to evaluate the characteristics of the serum ferritin level as an early screening test of KD-MAS.

## 2. Materials and Methods

### 2.1. Study Materials and Data Collection

We prospectively recruited patients with KD who were diagnosed with complete or incomplete KD at the Department of Pediatrics, Kyungpook National University Children’s Hospital and Keimyung University Dongsan Medical Center between September 2019 and June 2020. In January 2020, the first patient with SARS-CoV-2 infection occurred in Korea. A total of 56 patients were diagnosed with KD in 2020, and polymerase chain reaction (PCR) for SARS-CoV-2 was performed for all patients on the first day of hospitalization. There was no patient with evidences of current or recent SARS-CoV-2 infection (positive PCR, or COVID-19 exposure within 4 weeks prior to the onset of the illness).

Complete and incomplete KD was diagnosed according to the 2017 American Heart Association guideline [12]. Patients showing persistent or recrudescent fever >5 days and at least four of the five typical clinical manifestations (bilateral conjunctival injection, polymorphic rash, lip redness and strawberry tongue, palm and sole erythema/edema, and cervical lymphadenopathy) were diagnosed with complete KD. Patients showing fewer than four clinical manifestations were diagnosed with incomplete KD. We defined patients with refractory KD as those who had recrudescent or persistent fever at least 36 h after the end of initial intravenous immunoglobulin (IVIG) infusion.

To diagnose MAS, we adopted the HLH-2004 criteria [11] where at least five of the following eight criteria must be fulfilled: (1) fever; (2) splenomegaly; (3) cytopenia ≥ 2 cell lineages in the peripheral blood (hemoglobin < 9 g/dL, in infants < 4 weeks: hemoglobin < 10 g/dL, platelets < 100 × 103/μL, neutrophils < 1 × 103/μL); (4) hypertriglyceridemia (fasting, 265 mg/dL) and/or hypofibrinogenemia (<150 mg/dL); (5) hemophagocytosis in bone marrow, spleen, lymph nodes, or liver; (6) low or absent natural killer-cell activity; (7) ferritin levels > 500 ng/mL; and (8) elevated soluble CD25 (soluble IL-2 receptor ≥ 2400 U/mL). Patients in both groups received 2 g/kg IVIG as initial treatment for KD.

The total 158 children were enrolled and divided into two groups: KD (*n* = 153) and KD-MAS (*n* = 5).

### 2.2. Laboratory Test

We performed conventional and detailed laboratory tests on the first day of admission.

The following parameters were measured as the conventional laboratory test: white blood cell (WBC) count, percentage of neutrophil in WBC (% neutrophils), hemoglobin, platelet counts, aspartate aminotransferase (AST), alanine aminotransferase (ALT), serum total protein, albumin, erythrocyte sedimentation rate (ESR), C-reactive protein (CRP), and N-terminal pro-brain natriuretic peptide (NT-ProBNP). Detailed laboratory test included ferritin, triglyceride (TG), fibrinogen, and lactate dehydrogenase (LDH).

### 2.3. Echocardiography

The presence of coronary artery complications was checked by echocardiography by two pediatric cardiologists. We calculated the z-score from the coronary artery diameter and aortic valve annular diameter [13]. We obtained the luminal diameters from at least two points including the proximal right coronary artery and left main coronary artery. The aortic valve annular diameters were measured from parasternal long-axis views. Coronary artery dilatation was recognized when the z-score of the coronary artery size was >2.0. Coronary artery aneurysm was defined as a z-score of the coronary artery size >2.5 [12].

### 2.4. Statistical Analysis

All statistical analyses were performed using IBM SPSS Statistics for Windows, version 26.0 (IBM Co., Armonk, NY, USA). To compare the clinical characteristics and laboratory data, Mann–Whitney test and Chi-square test were performed. A *p*-value of <0.05 was considered statistically significant.

### 2.5. Ethics Statement

This study was reviewed and approved by the Institutional Review Board of Kyungpook National University Chilgok Hospital (approval No. KNUCH 2021-02-006).

## 3. Results

### 3.1. Clinical Characteristics

Table 1 shows the clinical characteristics of all patients. The median age, proportion of male patients, and total duration of fever were not significantly different between the groups (*p* = 0.568, *p* = 0.650, and *p* = 0.641, respectively).

Conjunctival injection was significantly more frequent in KD group (*p* = 0.035). Changes in the extremities, skin rash, the lips and oral cavity, cervical lymphadenopathy, and BCGitis, as well as the proportion of patients diagnosed with incomplete KD at the time of initial diagnosis, were not significantly different between the groups.

Hepatosplenomegaly was observed in two patients in the KD-MAS group but not in the KD group (*p* = 0.001).

IVIG resistance was significantly higher in the KD-MAS group than in the KD group (*p* = 0.017, Table 1). There was no significant difference in the frequency of coronary artery complication between groups (*p* = 1.000).

### 3.2. Laboratory Findings

The results of conventional and detailed laboratory tests of the two groups are summarized in Table 2 and Table 3.

ESR, total protein, and albumin were significantly lower in the KD-MAS group than in the KD group (*p* = 0.002, *p* = 0.004, and *p* = 0.001, respectively). Total bilirubin was significantly higher in the KD-MAS group than in KD group (*p* = 0.019). CRP and the level of NT-proBNP were not significantly different between the groups (*p* = 0.754, and *p* = 0.926, respectively).

Ferritin level was significantly increased in the KD-MAS group compared with KD group (*p* = 0.001, Table 3). In addition, there were significant differences as higher triglyceride and fibrinogen, and lower LDH in KD-MAS group than in KD group (*p* = 0.001).

### 3.3. Clinical Course of the KD-MAS Group

The clinical course of patients is described in Table 4.

Except one patient, patients did not meet typical clinical manifestations of KD and were diagnosed as incomplete KD.

The ferritin level was >500 ng/mL in all patients, and four patients (80%) showed hemophagocytosis based on bone marrow biopsy.

The time taken to final confirmation of MAS after KD diagnosis varied between 1 and 16 days. Although two patients below 1 year old needed time over 7 days to final diagnosis, other patients were finally diagnosed as MAS within 7 days.

Case 1 received further treatment with methylprednisolone pulse therapy (30 mg/kg/day for 3 days) and infliximab (5 mg/kg) due to prolonged fever. After administration of infliximab, MAS was diagnosed with fever and laboratory test changes. After being diagnosed with MAS, he was transferred to another hospital and lost to follow up. Cases 2 and 4 were diagnosed with MAS after IVIG administration, so HLH-2004 chemo-immunotherapy was started immediately. Case 3 received further treatment with methylprednisolone pulse therapy and diagnosed with MAS, so HLH-2004 chemo-immunotherapy was started. Case 5 had afebrile after 2nd IVIG administration; her condition then improved, and she was discharged. After discharge, MAS was diagnosed using laboratory findings, but no additional treatment was performed because laboratory findings were improved and patient was asymptomatic in follow up visit. All cases survived (except one patient was lost to follow-up) and finished the treatment safely.

## 4. Discussion

The present study demonstrated the following findings that can indicate KD combined with MAS on the first day of admission: (1) hepatosplenomegaly and (2) ferritin level at >500 ng/mL. Conventional laboratory test and typical clinical feature of KD were not helpful to distinguish between KD with and without MAS.

The mortality rate of MAS varied depending on the underlying diseases of the patients. According to previous studies, mortality rate of MAS ranges from 8% to 22% [14,15]. Mortality rate of KD-MAS was reported to be 13–25% [8,16,17], but reports of the mortality are still insufficient. In addition, the incidence of coronary artery complications in KD-MAS is high at 46%, which is much higher than in KD without MAS (15–25%) [16].

To reduce mortality rate and cardiac complication, early screening and diagnosis are important. In clinical practice, MAS tends to be under-recognized because the principal clinical features have not been established and overlaps with KD, which makes differential diagnosis challenging.

Persistent fever with splenomegaly has been suggested as the main indication in KD-MAS [5,17]. In another study, male sex, age over 2 year, incomplete KD, IVIG resistance, or persistent fever greater than 10 days were highly associated with the occurrence of MAS [6,18]. In the present study, gender and age did not differ between groups. Although it is not statistically significant, the total duration of fever was longer in the KD-MAS group than in the KD group. The incidence of conjunctival injection was significantly lower in the KD-MAS group than in the KD group, however, it was difficult to distinguish the two diseases by other principal clinical features of KD. Characteristically, hepatosplenomegaly was only shown in the KD-MAS group like the previous studies. Since KD is diagnosed with visible findings (conjunctival injection, the changes of hand/foot/lips/oral mucosa, and rash), hepatosplenomegaly can be overlooked in the initial diagnosis of KD. Its detection on the physical examination is important for screening and early diagnosis of MAS in patients with KD.

It is helpful to detect transition to MAS using laboratory biomarkers. KD-MAS showed thrombocytopenia, elevated liver transaminases, and marked hyperferritinemia compared with KD without MAS [8,19,20]. NT-proBNP was also suggested as diagnostic markers useful for early detection of MAS in KD [16].

Ferritin is one of the prominent diagnostic markers in MAS, and in diagnostic criteria in both HLH-2004 criteria and consensus criteria of MAS in sJIA [10,11,18]. The diagnostic cutoff value of ferritin level differs by 500 ng/mL from the HLH-2004 criteria and by 684 ng/mL from the sJIA-MAS 2016 criteria [10,11]. Ferritin levels ranging from 1500 to 60,590 ng/mL in patients with KD-MAS are much higher than the diagnostic cutoff value of HLH-2004 and sJIA-MAS 2016 criteria [4,7,8,16,21]. In addition, ferritin level has been an important predictor of nonresponsiveness to initial IVIG therapy in KD and disease severity in sepsis [22,23]. Interestingly, in the present study, ferritin level was already significantly high in the KD-MAS group from the first day of admission, and KD patients with normal ranges of ferritin levels did not show elevation of ferritin from admission to discharge. We thought that confirmation of hyperferritinemia from the first day of admission and close follow-up was helpful in early diagnosing MAS in patients with KD.

It is already known that hyperbilirubinemia can occur in both KD and MAS [4,24], but reports comparing degrees of hyperbilirubinemia between KD and KD-MAS are still insufficient. The present study showed an increase in total bilirubin in the KD-MAS group compared to the KD group. These results support that increased total bilirubin in KD patients may be considered a predictor of MAS transition from KD. LDH is also known to be increased in both KD and MAS [7,25]. In present study, LDH increased significantly in KD-MAS from the first day of hospitalization, so increased LDH level should be considered as a predictor of MAS transition from KD. Triglyceride and fibrinogen are important laboratory markers included in the MAS diagnostic criteria [10,11]. In present study, the KD-MAS group patients showed significant increase in triglyceride and decrease in fibrinogen from the first day of hospitatlization. In tracking the transition from KD to KD-MAS, these laboratory markers may be helpful in addition to hyperferritinemia.

The present study showed that it took between 1 and 16 days to finally confirm MAS after KD diagnosis. Laboratory follow-up is required to fulfill MAS diagnostic criteria because laboratory results conflicted. If KD-MAS is suspected from the first day of hospitalization, close monitoring can shorten the time to final diagnosis for KD-MAS. Among our patients, there was even a patient who was able to diagnose KD-MAS on the first day of admission. Two patients under the age of 1 took longer to diagnose KD-MAS than those over the age of 2, even though they required close monitoring. It might be related that patients under the age of 1 take longer to diagnose KD because of deficiency of typical clinical features.

The previous studies revealed that KD-MAS was highly unresponsive to IVIG. In KD, approximately 10–20% of patients faiedl to respond to primary IVIG treatment [7,26,27,28,29]. Patients with KD-MAS had very high rates of IVIG resistance from 50% to 90% [4,9,27]. The present study showed similar result about rates of IVIG resistance like the previous studies.

The main limitation of the present study is its small sample size, especially the KD-MAS group, thus affecting the statistical results. In addition, the statistical assessment of the effect of early ferritin screening for morbidity and mortality KD-MAS was difficult.

## 5. Conclusions

In conclusion, early ferritin screening test is important in patients with principal clinical features that may suspect KD. In the present study, we included ferritin level in the primary laboratory test to differentiate between KD with and without MAS early, but considering low incidence of KD-MAS, we recommend the ferritin test in KD patients with hepatosplenomegaly, abnormal results of biomarkers (low ESR/fibrinogen, high total and bilirubin/triglyceride/LDH), or refractoriness of initial IVIG treatment.

## Figures and Tables

**Table 1:** Comparison of clinical characteristics between groups.

	KD (*n* = 153)	KD-MAS (*n* = 5)	*p* Value
Age (months)	35.0 (15.5–49.5)	26.0 (1.0–62.5)	0.568
Male sex	89 (58.2)	2(40.0)	0.650
Total duration of fever (days)	6 (5–8)	8 (2–19)	0.641
Conjunctival injection	107 (69.9)	1(20.0)	0.035
Extremity change			
Erythema of palms and soles	76 (49.7)	1 (20.0)	0.368
Skin rash	97 (63.4)	2 (40.0)	0.363
Changes of the lips and oral cavity	98 (64.1)	2 (40.0)	0.357
Cervical lymphadenopathy	70 (45.8)	3 (60.0)	0.663
BCGitis	44 (28.8)	1 (20.0)	1.000
Hepatosplenomegaly	0 (0.0)	2 (40.0)	0.001
Incomplete KD	102 (66.7)	4 (80.0)	1.000
IVIG resistance	66(43.1)	5(100.0)	0.017
Coronary artery complication	32(21.0)	1(20.0)	1.000

Values are presented as numbers (%) or median and range. Abbreviations: KD, Kawasaki disease; MAS, macrophage activation syndrome; BCG, Bacille de Calmette-Guerin; IVIG, intravenous immunoglobulin.

**Table 2:** Conventional laboratory findings of two groups on the first day of admission.

Laboratory Findings	KD (*n* = 153)	KD-MAS (*n* = 5)	*p* Value
WBC (103/μL)	13.1 (10.3–17.7)	13.4 (4.8–18.1)	0.548
Neutrophil (%)	65.7 (57.0–78.4)	65.5 (48.5–73.3)	0.617
Hemoglobin (g/dL)	11.5 (11.1–12.0)	10.8 (10.4–11.4)	0.050
Platelet (103/μL)	358.0 (297.0–421.5)	262.0 (103.5–374.5)	0.065
CRP (mg/dL)	4.9 (2.9–8.8)	12.0 (0.3–20.2)	0.754
ESR (mm/h)	70.0 (43.5–91.5)	9.0 (3.5–45.3)	0.007
Total protein (g/dL)	6.6 (6.2–7.0)	5.8 (5.3–6.1)	0.004
Albumin (g/dL)	4.0 (3.9–4.3)	3.4 (3.1–3.7)	0.001
AST (U/L)	38.0 (27.0–77.5)	283.0 (42.5–408.5)	0.051
ALT (U/L)	26.0 (15.0–110.5)	188.0 (18.5–575.0)	0.128
Total bilirubin (mg/dL)	0.4 (0.3–0.6)	3.2 (0.5–6.6)	0.019
Sodium	136.0 (134.0–138.0)	133.0 (132.5–137.5)	0.177
NT-proBNP (pg/mL)	466.5 (210.3–1079.8)	638.5 (244.8–709.8)	0.926

Values are presented as median and range. Abbreviations: WBC, white blood cell; ESR, erythrocyte sedimentation rate; CRP, C-reactive protein; AST, aspartate transaminase; ALT, alanine transaminase; LDH, lactate dehydrogenase; NT-proBNP, N-terminal pro-brain natriuretic peptide.

**Table 3:** Detailed laboratory findings of two groups on the first day of admission.

Laboratory Findings	KD (*n* = 153)	KD-MAS (*n* = 5)	*p* Value
Ferritin (ng/mL)	135.0 (96.2–204.5)	2529.0 (820.5–14,455.5)	0.001
Triglyceride (mg/dL)	99.0 (72.5–131.5)	269.0 (182.5–853.5)	0.001
Fibrinogen (mg/dL)	555.5 (458.8–640.3)	252.5 (177.6–293.7)	0.001
LDH (U/L)	384.0 (256.0–561.5)	1319.9 (748.0–1485.0)	0.001

Values are presented as median and range. Abbreviations: LDH, lactate dehydrogenase; n, number; KD, Kawasaki disease; MAS, macrophage activation syndrome.

**Table 4:** Clinical and laboratory profiles of patients with KD-MAS.

Patient Number	1	2	3	4	5
Gender/age(years)	M/2	F/5	F/4	M/0	F/0
Fever duration (days)	14	8	24	2	2
KD type	Incomplete	Incomplete	Complete	Incomplete	Incomplete
Hepatosplenomegaly	N	N	Y	Y	N
Hemophagocytosis	Y	Y	Y	Y	N
Hemoglobin (g/dL)	10.4	11.3	11.5	10.3	10.8
Platelet (10^3^/μL)	312	106	262	437	101
ESR (mm/h)	61	8	10	2	57
AST (U/L)	56	492	283	29	325
AST (U/L)	19	817	333	18	188
Total bilirubin (mg/dL)	0.34	3.2	0.57	5.99	7.28
Total protein (g/dL)	6.4	5.8	5.8	5.3	5.2
Albumin (g/dL)	3.3	3.4	3.6	3.7	2.8
Ferritin (ng/mL)	590	2529	12411	16500	1051
Triglyceride (mg/dL)	188	243	295	66	164
Fibrinogen (mg/dL)	220	157	303	108	265
LDH (U/L)	1784	748	1485	2636	823
Coronary artery complication	N	N	N	N	Y
Timing for final diagnosis from KD to MAS	6 days	3 days	1 days	11 days	16 days
Treatment	IVIG MP pulse Infliximab	IVIG HLH-2004	IVIG MP pulse HLH-2004	IVIG HLH-2004	IVIG (2 times)
Outcome	Transfer to another hospital	Survived	Survived	Survived	Survived

Abbreviations: KD, Kawasaki disease; MAS, macrophage activation syndrome; Y, yes; N, no; ESR, erythrocyte sedimentation rate; AST, aspartate transaminase; ALT, alanine transaminase; LDH, lactate dehydrogenase; IVIG, intravenous immunoglobulin; MP. methylprednisolone; HLH, hemophagocytic lymphohistiocytosis.

## Data Availability

Data from this study can be obtained by request to the authors.
